# Feasibility of Using Electrical Impedance Spectroscopy for Assessing Biological Cell Damage during Freezing and Thawing

**DOI:** 10.3390/s21124129

**Published:** 2021-06-16

**Authors:** Sisay Mebre Abie, Ørjan Grøttem Martinsen, Bjørg Egelandsdal, Jie Hou, Frøydis Bjerke, Alex Mason, Daniel Münch

**Affiliations:** 1 Department of Physics, University of Oslo, 0316 Oslo, Norway; s.m.abie@fys.uio.no (S.M.A.); Jieho@fys.uio.no (J.H.); 2 Department of Clinical and Biomedical Engineering, Oslo University Hospital, 0372 Oslo, Norway; 3 Faculty of Chemistry, Biotechnology and Food Science, Norwegian University of Life Sciences, 1432 Ås, Norway; bjorg.egelandsdal@nmbu.no; 4 Animalia, Norwegian Meat and Poultry Research Centre, 0513 Oslo, Norway; froydis.bjerke@animalia.no (F.B.); alex.mason@nmbu.no or or daniel.munch@nmbu.no (D.M.); 5 Faculty of Science and Technology, Norwegian University of Life Sciences, 1432 Ås, Norway; 6 Faculty of Ecology and Natural Resource Management, Norwegian University of Life Sciences, 1432 Aas, Norway

**Keywords:** bioimpedance, freezing, thawing, meat, LSTM-RNN, machine learning

## Abstract

This study was performed to test bioimpedance as a tool to detect the effect of different thawing methods on meat quality to aid in the eventual creation of an electric impedance-based food quality monitoring system. The electric impedance was measured for fresh pork, thawed pork, and during quick and slow thawing. A clear difference was observed between fresh and thawed samples for both impedance parameters. Impedance was different between the fresh and the frozen-thawed samples, but there were no impedance differences between frozen-thawed samples and the ones that were frozen-thawed and then stored at +3 °C for an additional 16 h after thawing. The phase angle was also different between fresh and the frozen-thawed samples. At high frequency, there were small, but clear phase angle differences between frozen-thawed samples and the samples that were frozen-thawed and subsequently stored for more than 16 h at +3 °C. Furthermore, the deep learning model LSTM-RNN (long short-term memory recurrent neural network) was found to be a promising way to classify the different methods of thawing.

## 1. Introduction

Freezing is one of the most widely used methods of meat preservation [1,2]. Around 50–75 percent of the weight of meat is water. When water freezes, it expands, and ice crystals can cause extensive damage to cell membranes and other structures. This means that besides its obvious advantages, freezing can also cause cell and tissue damage, often resulting in poorer meat quality. Before consumption, frozen meat must be thawed, with additional effects on meat quality, e.g., by recrystallization during phase transition. Typical detrimental effects of freezing and thawing on meat quality are thaw loss (TL), and changes to meat color and tenderness [3,4,5,6,7]. Therefore, identifying optimum thawing procedures can reduce quality and result in economic loss.

Surprisingly, and in contrast to freezing, there is no clear scientific consensus on how thawing rate affects meat qualities. Some authors report that quicker thawing, for example in a water-bath, reduces drip (‘thaw’) loss (as reviewed by Leygonie et al. [2]). However, Gonzales-Sanguinetti et al. [8] demonstrated that quick thawing resulted in more drip loss, whereas Ngapo et al. [4] reported lower drip loss with rapid thawing. Linares et al. [9] explained that, in the case of quick thawing, the fluid released from fibers cannot be reabsorbed and, as a result, there is higher drip loss. On the other hand, slow thawing can promote ice recrystallization, which may lead to increased drip loss.

Thawing and freezing are inverse processes. They are different in the phase change direction, cooling and heating process, as well as in phase transition time and internal temperature variations [10,11]. The thawing process causes the ice to melt inside the food, which mostly absorbs the resultant water. The freshness of the food is subsequently restored to a level that is, ideally, close to before being frozen. Various studies suggest that different biological materials require different thawing rates. For instance, for good quality frozen vegetables, bread and pastries, quick thawing is required, but for fish and meat, slow thawing is better if overheating is critical [12].

Thawing practices include traditional atmospheric thawing, water immersion thawing and, more recently, microwave or high-pressure assisted thawing [13]. Again, in contrast to rapid freezing, the benefits of rapid thawing for reducing drip loss are much less clear, possibly due to missing internal temperature profiles for the diverse food products.

In this study, we investigated bioimpedance (BI) as an analytical tool in food-related contexts and measured electrical bioimpedance of pork meat in the frozen state and throughout the thawing process. Freeze- and thaw-related damage to cell membranes and other muscle tissue structures also affects the electrical properties of meat. Electrical impedance testing has been shown to be a relatively simple, rapid, and inexpensive way of detecting and assessing such damage [14,15,16]. More generally and apart from damage, bioimpedance testing of biological tissues at low stimulus frequencies mostly reflects changes in extracellular fluid fractions (‘thaw channels’), as intact membranes impede the stimulus current flow [17]. At higher frequencies, however, the current can pass through capacitors (e.g., membranes) and impedance values reflect both fluid fractions in the intra- and extracellular compartments.

The impedance is represented by a complex number which has both a magnitude and a phase angle. The magnitude of the impedance is $Z=\sqrt{R^{2}+X^{2}}$ and the phase angle is φ=arctan(X/R), where *R* is the real part, resistance, and *X* is the imaginary part, reactance, of the impedance. The resistance is mainly caused by free ions, whereas the reactance is primarily caused by capacitive effects at the cell membranes.

To better understand the impedance of pork meat subjected to freezing, and to further demonstrate the feasibility of the bioimpedance technique for monitoring thawing, this study is focused on measurements of electrical impedance of pork meat during different thawing procedures.

The relative changes in the impedance of pork samples during storage were determined by the following equation:

(1) $$Z\left(\%\right)=(Z_{0}+Z_{i})/Z_{0}$$

where *Z* is the relative change in impedance (%), *Z*_0_ is the impedance of the original sample (day 0), and *Z_i_* is the impedance of samples at a given storage interval [18].

The electrical impedance of biological tissues varies widely because of their diverse electrical properties. This diverse electrical nature is due to the fact that the electrical properties of tissue depend on their inhomogeneity, anisotropy, composition, structure, physiological state, electrode polarization, frequency, moisture, and temperature.

According to different studies, impedance decreases between the beginning of freezing and the end of thawing. These changes in impedance may serve as an important tool to quantify the properties of the sample. For instance, Yu et al. [14] and Kent et al. [19] measured impedance of fish fillets at 100 Hz and microwave frequency, respectively. Both authors obtained a significant impedance difference between the beginning and the end of freezing and thawing. Yu et al. [14] found that monitoring impedance changes during various freezing and heating conditions offered an alternative way of monitoring damage to the biomaterial.

Srivastava et al. [16] tested sea bass samples and measured the impedance of red blood cells (RBC) in different freezing and thawing conditions to detect the hemolysis of RBCs after thawing. They suggested that the reactance (X) spectra at frequencies higher than 500 kHz were particularly suitable to distinguish different freezing histories in sea bass samples (compare also [20]). Yu et al. [21] suggested that dynamic low frequency impedance measurements could provide useful information for ice formation and thawing, which is critical information to assess the freezing damage of biological materials. They also suggested the impedance change rate (ICR) as an alternative method to evaluate the freezing behavior of biological materials, especially around the phase-change region. It exhibits a sudden drop in ICR when the phase change occurs and the latent heat is released, possibly revealing the relation between ice formation and cell damage.

To the best of our knowledge, there are no reports regarding the use of electrical impedance technology for the rapid discrimination of differently thawed pork meat subjected to the same freezing. We will therefore assess if bioimpedance can detect differences in quality attributes linked to quick and slow thawing schemes.

## 2. Materials and Methods

### 2.1. Experimental Setup

Pork loin cuts were obtained from a slaughterhouse at three days postmortem and sliced to individual samples 5 cm *×* 5 cm *×* 4 cm in size, with a weight of 180 ± 30 g. All possible efforts were made to make all samples the same size (effects of weight variation can be assessed by thaw and drip loss measurement). The pieces were packed in a polyethylene bag by a 99% vacuum packaging method. The samples were placed in the bag so that the electrodes (impedance probe) could be inserted in the cut face of the loin slices. The four-electrode bioimpedance probe was made of pin-shaped, gold plated, copper electrodes, with a length and diameter of 17.8 mm and 0.63 mm, respectively. The distance between the pins of the tetrapolar probe was 1.1 – 2.2 – 1.1 cm (compare Supplemental Material Figure S1 for an image of the measuring probe). Then, the temperature probe (offset slightly from the center of the middle two electrodes) was inserted at a depth of 20 mm. We used duct tape to fix the probes and cable ties to avoid pulling and snagging of the leads. Ten samples were assigned for fast thawing (water-bath) and ten samples for slow thawing (air thawing), respectively. A Zurich Instruments MFLI (Zurich Instruments AG, Switzerland) was used to measure resistance (R) and reactance (X) during the thawing processes, using an applied voltage of 400 mV rms and 36 frequencies from 10 Hz to 510 kHz. The samples were measured every three minutes while the temperature increased to −15 °C and, thereafter, every five minutes until it reached 0 °C. From 0 °C to 4 °C, the measurements were carried out for each 0.5 °C.

After measurements of electric bioimpedance in the fresh (unfrozen state), all samples were kept at −85 °C for one month. Then, the frozen meat samples were individually thawed under two different conditions:

- Quick water-bath (QWB): very quick, one-step-thawing. Frozen samples were directly transferred into a 10 °C water-bath until the core temperature reached 4 °C. During this process, a 68-litre plastic crate was used as water container. The sample had to be anchored using a cord attached to the bottom of the outer bag and fed through the bottom of the crate since attempts with up to 5 kg weights were not sufficient to keep the samples submerged. The samples were then stored in the cooling room with a temperature between 2 and 4 °C.
- Slow air: slow one-step thawing. Frozen samples were transferred to an ambient air temperature of 10 °C until the core temperature reached 4 °C. Then, samples were stored in the cooling room with a temperature between 2 and 4 °C. During this process, the sample hung from a horizontal bar that was prepared for this purpose.

During the thawing, the temperature at the center of the meat sample was recorded. Thawing was considered complete when the final core temperature was ~4 °C. After thawing, samples were stored at 2–4 °C for more than 16 h before measuring final impedance and meat thawing loss.

### 2.2. Statistical Analysis

The use of the entire spectrum is often not necessary since there is a strong correlation between impedance values at adjacent frequencies [17]. Therefore, the *P_y_* parameter, which is the direct measure of the beta dispersion and cell membrane-related responses, was used in the statistical analysis to assess the damage due to the two different thawing processes. According to Pliquett et al. [22] the *P_y_* parameter can be calculated as:

(2) $$P_{y}=\frac{\left(R_{0}-R_{inf}\right)100}{R_{0}}$$

where resistance values at lower (*R*_0_) and higher frequencies (*R_inf_*) are derived from Cole fitted data. Generally, *P_y_* reflects the abundance of current impeding structures, e.g., membranes, with lower and higher *P_y_*-values indicating low and high cell and membrane densities, respectively [22]. Statistical treatment of the data was performed using the MATLAB statistical toolbox. Mann-Whitney U test (MWU) statistics were calculated to assess possible differences between the experimental groups.

Python-based artificial neural network analysis was used for discriminating the samples according to treatment group based on the impedance measurement results. Due differences in geometry between the samples, the absolute value of impedance or impedance change may vary for different samples of the same materials. Thus, to quantify the damage to the tissue as a consequence of thawing, the electrical measurement results were presented by indices (ratios) between the reference measurements (unfrozen, fresh state, “*un*”) and the measurements after freezing and thawing (“*ft*”) according to Equations (3) and (4) [23].

(3) $$R:index=\left(\frac{R_{un}-R_{ft}}{R_{un}}\right)$$

(4) $$X:index=\left(\frac{X_{un}-X_{ft}}{X_{un}}\right)$$

where *R_un_*, *X_un_*, *R_ft_* and *X_ft_* are resistance and reactance unfrozen (fresh), and resistance and reactance of fast thawed (water-bath thawed), respectively. The results presented in this way were used in artificial neural network analysis to test the difference between fast and slow thawed samples (water-bath thawed or air thawed) in electrical impedance. The impedance measurements (module and phase of impedance for each frequency) were considered dependent variables and treatment temperature (from 0 °C up to 4 °C) was the factor in these analyses.

## 3. Results

**Figure 1:**
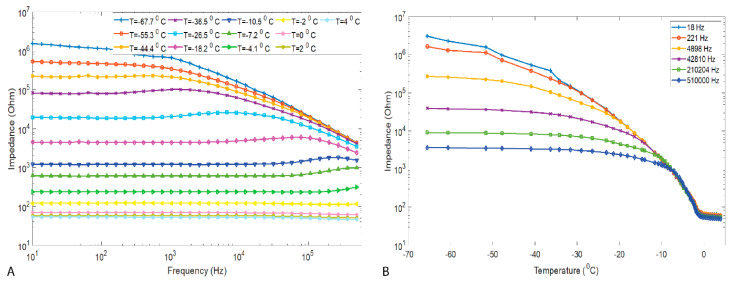
The (**A**) typical plots showing the impedance of the meat during the described thawing process, and the (**B**) typical plots of impedance vs. temperature for six selected frequencies. The impedance measurements were taken during the fast thawing (water-bath) of the pork muscle.

The electrical impedance of the meat was measured to examine the feasibility of using electrical impedance spectroscopy to monitor the thawing processes and to assess the potential of electrical impedance to differentiate the thawing methods. Figure 1A shows the typical meat impedance measured during fast thawing. The results indicated that, during the thawing process, the impedance decreased quickly with increasing temperatures, both during thawing and above the freezing point. The rate at which the impedance decreases was reduced when the temperature inside the sample approached its freezing points.

The impedance plots in Figure 1A show different curves, the top one for very low temperature (−68 °C) and the lower one for 4 °C, which is just above the freezing point, confirming that meat impedance is temperature dependent. In fact, not much is known about the bioelectrical properties of meat in the frozen state and how they depend on temperature (when frozen) and how they change during phase transition from frozen to defrosted. Figure 1B demonstrates the relation between temperature and electrical impedance at six selected frequencies for temperatures from −68 °C up to 4 °C. For the frequencies 510,000 Hz, 201,402 Hz, and 42,810 Hz the impedance remained relatively constant until the temperature reached around −20 °C and then it started to decrease. From around −10 °C, the decrease was more drastic up to −2 °C. For the lower frequencies 18 Hz and 221 Hz, the impedance slowly decreased from −68 °C up to −35 °C, with a more drastic decrease above −35 °C until −2 °C. In general, based on these findings, the impedance decreases with temperature increase. In temperature ranges dominated by phase transition (−12 °C to −1.5 °C), the impedance decreased very rapidly at all frequencies, whereas in the dominating temperature increase zone (<−18 °C and >−1.5 °C), the impedance change was slower.

**Figure 2:**
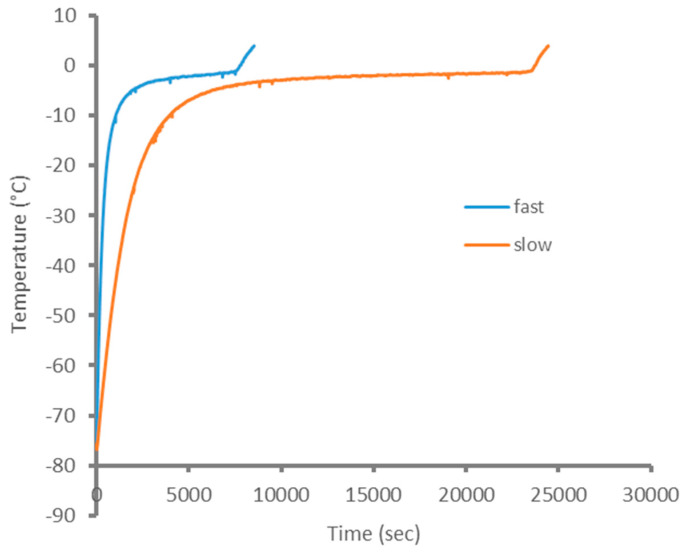
Typical thawing curve for meat during air and water-bath thawing. The temperature was measured using a K-type thermo-logger with the probe tip located at the center of the pork muscle.

Figure 2 shows the representative temperature curves for the two thawing processes used. During thawing, the temperature increased quickly up to about −10 °C, i.e., within a temperature range where phase transition, ice melting, in the meat begins. The temperature curve then flattened with further thawing, due to the large amount of latent heat that is needed for melting. At around −10 °C, when thawing was completed, the applied heat again led to a more rapid temperature increase in the meat samples. In conclusion, temperature curves in Figure 2 confirm that the two thawing treatments we used here do result in marked thawing rate differences, with the most rapid thawing induced by the water-bath treatment.

To investigate the effect of the thawing methods on the impedance response just after thawing, we used electric impedance of the last measurement of the thawing process when the core temperature had reached 4 °C. The possible challenges with this approach were that, while core temperature was similar in both treatment groups, the average temperature throughout the sample was possibly higher in the water-bath thawing group. It is known that the temperature change depends on the thermal properties of the tissue. Unfortunately, there is not enough information about the thermal properties of the tissue (specific heat capacity, thermal conductivity, and heat diffusivity), which prevents us from accurately predicting the temperature distribution of the meat samples.

**Figure 3:**
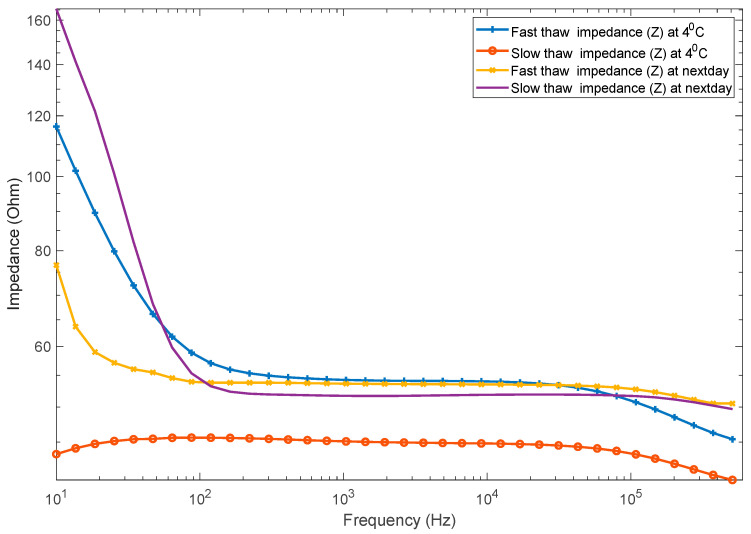
The impedance of the meat for water-bath (fast) thawing and air (slow) thawing processes at 4 °C and the next day. Each line represents the mean impedance of ten meat samples.

As Figure 3 demonstrates, the fast and slow thawing processes showed a difference in impedance measured at 4 °C, which was the end temperature of both thawing processes. However, that difference decreased by a large amount when measured the next day. In particular, mean impedance magnitude of slow thawed meat increased in the next day data, and the difference was particularly large at the lower frequency range. This could be due to extensive cell structure or membrane destruction and a loss of most conducting fluids and ions in the slow thawed samples.

Furthermore, to explore the effect of the thawing methods on the impedance response of the meat, the P_y_ parameters for each impedance measurement, which were taken at every 0.5 °C temperature difference between 0 °C and 4 °C during thawing as well as in the next day, were calculated. An MWU statistical analysis was performed on these P_y_ parameters for each temperature to compare possible cell damage.

The results in Table 1 show that bioimpedance was capable of detecting the difference between the two thawing processes. For instance, at 4 °C the average P_y_ value of water-bath thaw was significantly different from the average P_y_ value of air thaw with a p-value less than 0.01. On the contrary, for electrical impedance measurement at the final day (the next day after thawing completed), the average P_y_ value of water-bath thaw was not significantly different from the average P_y_ value of air thaw with a *p*-value of 0.3107.

**Figure 4:**
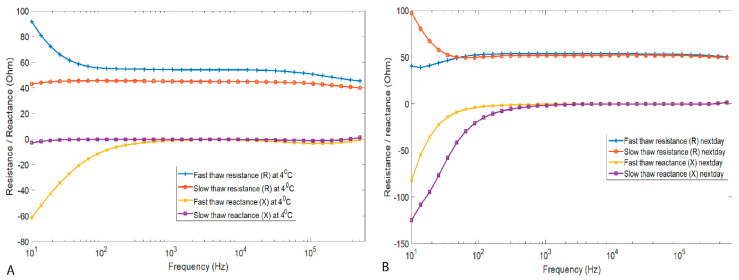
The (**A**) impedance components resistance (R) and reactance (X) of pork meat tissue at 4 °C after freezing and thawing, and the (**B**) resistance and reactance curves of slow thawed and fast thawed pork meat tissue. Each line in both figures represents the mean impedance of ten meat samples.

To assess how the thawing method affected the impedance response in defrosted and stored pork loin muscle, we compared the impedance values, which were measured from the samples stored for more than 16 h after thawing. For these measurements, the samples were stored in the chilling room at 2–4 °C. From the graphical representation of these measurements, shown in Figure 4B, the resistance was higher at low frequencies (10–100 Hz) for air thawing and no observable treatment effect could be seen for resistance above 100 Hz. Similarly, the reactance was frequency dependent in the low frequency region and became frequency independent above 1 kHz for both thawing methods.

As shown in Figure 4A, the resistance of water-bath thawed meat decreased and the reactance increased in the low frequency region until 200 Hz, and became constant from 200 Hz up to 70 kHz. The resistance again started to decrease above 70 kHz and the reactance increased. On the contrary, the resistance and the reactance of the slow thawed meat was almost constant throughout the whole frequency spectrum.

To test the difference between fast and slow thawed samples in electrical parameters (resistance, reactance, and phase angle), MWU statistics were calculated for the entire spectrum of all the impedance data collected above 0 °C. The results showed that resistance had a statistical difference between the groups at high frequencies. Reactance had a statistical difference at lower and higher frequencies, but the phase angle had a statistical difference at most of the measured frequencies.

Furthermore, to assess the feasibility of the impedance spectroscopy technique in the differentiation of water-bath thawed and air thawed meat, discrimination analysis was performed to differentiate (classify) the samples into their respective groups based on the electrical impedance measurement result. According to Li et al. [15], machine learning showed its strength in the classifications of chicken breasts with different melting times, when it was used in the analysis of passive electrical properties of tissues. These kinds of decision making and interpreting processes can be done without explicit human instructions, as the model will pick up the underlying patterns and use the learned features to make a prediction on a new impedance data set accurately and automatically.

Long short-term memory (LSTM) artificial recurrent neural networks were chosen to classify different thawing treatment groups, as this type of network can carry historical information across its layers. Various architectures were tested, with different numbers of LSTM layers and hidden units. The resulting model included two LSTM layers with batch normalization and drop out between the layers. To translate the probabilistic nature of the model into either fast or slow thawing categories, a dense layer with softmax as the activation function was applied after the LSTM layers. The training and testing data were split by 80% and 20%. Hyperparameters including batch size, ridge regularization, learning rate, RNN units, and dropout were tuned.

Two models were trained and tested, one using R as the input data and the other using X. There were 180 samples for each of the models, where 90 samples were from fast thawing measurements and 90 samples were from slow thawing measurements. Thirty-six frequency points, ranging from 10 Hz to 500 kHz, were included in each sample. The resultant model had a higher degree of differentiation of the water-bath thawing treatment group from the air thawing treatment group, with a test accuracy of 95% using X as input data and 91.67% accuracy using R as input data (Table 2). The algorithm was able to learn the differences between water-bath thawing and air thawing even when the number of data sets were very small, as in this case. However, for complex processing and accuracy, more experimental data are needed. Test accuracy was better using reactance data due to a bigger difference in amplitude at higher frequencies. This is plausible since destruction of cell membranes will influence the capacitance and, hence, the reactance to a large extent.

Drip loss is not only economically detrimental, but can give rise to an unpleasant appearance and result in a loss of soluble nutrients. Drip loss during thawing is caused by irreversible damage during the freezing, storage (recrystallization), and thawing processes [24]. Drip loss measurement involves the weighing of the meat at the start and the end of the process, and is expressed as a percentage of the initial weight. In our case, after thawing was completed, the sample was stored in the cooling room at 2–4 °C for a period of 16–26 h. Due to this time difference, the normalization with time was performed, and then a two-sample *t*-test statistical analysis was performed where the level of significance was set at *p* < 0.05. The result showed that there was no significant difference in the drip loss between the water-bath and air thawed meat after 16–25 h.

## 4. Discussion

Producers are typically mostly interested in how thawing affects the drip loss and, thus, moisture content of a product. Hence, from the results, it was shown that electrical impedance spectroscopy is a reliable method to differentiate the various thawing methods. In electrical impedance spectroscopy for real-time measurements of the impedance spectra of meat, the cell membrane behaves like a capacitor and acts as an insulator at low frequencies. At high frequencies, however, all capacitors are short-circuited. In fresh meat, the membranes are relatively intact, leading to high impedance, but in frozen-thawed samples, a considerable number of cell membranes are destroyed. This leads to reduction of the capacitive component of the samples and, together with an increase in the number of free electrolytes in the tissue, an overall decrease in the absolute value of the impedance is produced. In general, the freezing and thawing of meat further disrupts the cell membranes and the so-called beta-dispersion disappears [17]; hence, fresh meat can easily be distinguished from previously frozen meat.

The absence of impedance differences between fast thawed and slow thawed for the samples that were stored for more than 16 h at +3 °C could be the consequence of cell membrane disintegration during storage after defrosting. Specifically, differences due to different thawing treatment may be only transient and vanish, e.g., with further membrane disruption and fluid displacement early after defrosting.

The overall results showed that reactance can distinguish fast thawed from slow thawed meat samples at higher and lower frequencies. Resistance can only distinguish the fast from slow thawed sample in the high frequencies, and the phase angle can distinguish the fast from slow thawed sample in the medium frequency range.

Temperature was one of the factors that influenced the electrical measurements. The fast and slow thawing processes had different temperature change rates and different defrosting or decrystallization rates and, thus, thawing conditions affected the number of exudates by giving more or less time for extracellular water reabsorption [6]. During thawing, some of the water is not incorporated inside the cells and is lost as drip. The P_y_ parameter is a direct measure of the beta dispersion, and the integrity of cell membranes is a major factor influencing drip loss. Thus, if the beta dispersion is a direct measure of membrane integrity, it should also predict the drip loss. Since less capacitors (insulating cell membranes) are present, one would expect an extraordinarily high drip loss. Both the impedance result (P_y_ parameters) and the drip loss showed no significant difference between the two thawing processes. In conclusion, as the impedance cannot differentiate between the two thawing processes after 16 h of chilling storage, it is likely that P_y_-related quality factors (drip and tissue integrity) were not affected by slow vs. rapid thawing in our study.

## 5. Conclusions

Freezing and thawing are important processes in the food industry. Freezing is applied to food to retain quality and freshness, and to inhibit microbial growth. Thawing is also usually applied to any frozen food before consumption. This research was carried out by assessing characteristics of frozen pork muscle by different thawing methods (air at 10 °C and water at 10 °C). This study suggested that meat thawed by water-bath had a P_y_ value indicating more intact cell membranes than by air thawing. Thus, the impedance can differentiate between the two process. However, the impedance cannot differentiate between the two thawing processes after 16 h of chilling storage. In the chilling storage, the meat can lose all the integrated cell membranes that reduce the magnitude of the impedance.

We found that different thawing methods for pork meat gave no significant differences in the mean values of drip loss. Furthermore, impedance was found to drop quickly during the thawing process, but the impedance change ratio (ICR) varied between the samples due to differences in size and other characteristics. This may indicate that ICR could potentially be an interesting parameter to assess the degree of damage after the thawing of frozen meat.

## Figures and Tables

**Table 1:** Comparison of the P_y_ of the value of the impedance result measured during the fast and slow thawing processes.

Temperature (°C)	Group	Count	Median	Mean	St	IQR	MWU/*p*-Value
0	Fast thaw	10	16.7	16.9	3.81	3.97	0.0113
	Slow thaw	10	9.84	11.7	4.22	5.62	
0.5	Fast thaw	10	16.8	16.9	3.69	3.97	0.0113
	Slow thaw	10	9.95	11.7	4.09	5.44	
1	Fast thaw	10	16.7	16.8	3.62	4.08	0.0113
	Slow thaw	10	9.92	11.5	3.97	5.30	
1.5	Fast thaw	10	17.2	16.9	3.64	4.27	0.0073
	Slow thaw	10	9.86	11.4	3.88	5.03	
2	Fast thaw	10	16.9	16.8	3.70	4.36	0.0058
	Slow thaw	10	9.65	11.2	3.81	4.94	
2.5	Fast thaw	10	16.6	16.7	3.77	4.44	0.0073
	Slow thaw	10	9.47	11.0	3.68	4.68	
3	Fast thaw	10	16.4	16.5	3.82	4.49	0.0073
	Slow thaw	10	9.34	10.7	3.56	4.43	
3.5	Fast thaw	10	16.1	16.3	3.81	4.53	0.0073
	Slow thaw	10	9.17	10.5	3.47	4.16	
4	Fast thaw	10	15.9	16.1	3.90	4.48	0.0091
	Slow thaw	10	8.97	10.2	3.35	3.87	
Next day *	Fast thaw	10	4.57	4.65	1.85	2.23	0.3847
	Slow thaw	10	3.80	3.41	3.27	2.89	

* Next day indicates that the measurement was taken on the meat samples stored for more than 16 h in a temperature between 2 and 4 °C after thawing completed at 4 °C.

**Table 2:** Machine learning analysis results from resistance (R) and reactance (X).

	Using R	Using X
Test Accuracy	0.9167	0.95
Test Loss	0.3549	0.6233

## Data Availability

Not applicable.

## Supplementary Materials

The following are available online at https://www.mdpi.com/article/10.3390/s21124129/s1, Figure S1: Photo showing the tetrapolar impedance probe. Distances between the individual pins are 1.1 cm, 2.2 cm, and 1.1 cm, respectively.
