# Supplementary material for: Feasibility of Using Electrical Impedance Spectroscopy for Assessing Biological Cell Damage during Freezing and Thawing
Source: Sensors (Basel). 2021 Jun 16;21(12):4129. doi: 10.3390/s21124129 (PMC8235392; doi:10.3390/s21124129)
Supplement: Supplementary file 1 [file sensors-21-04129-s001.zip › sensors-1183876-supplementary.pdf]

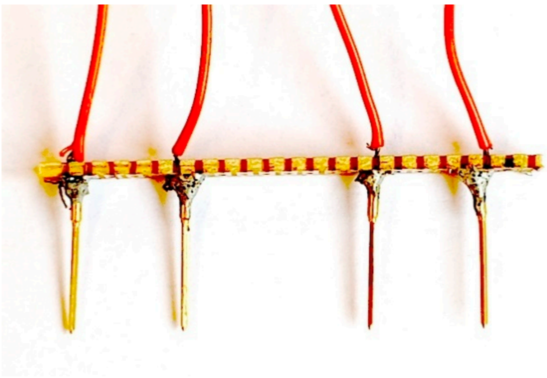

Figure S1. Photo showing the tetrapolar impedance probe. Distances between the individual pins are 1.1 cm, 2.2 cm, and 1.1 cm, respectively.
